# Supplementary material for: A Clinically Selected Staphylococcus aureus clpP Mutant Survives Daptomycin Treatment by Reducing Binding of the Antibiotic and Adapting a Rod-Shaped Morphology
Source: Antimicrob Agents Chemother. 2023 May 15;67(6):e00328-23. doi: 10.1128/aac.00328-23 (PMC10269151; doi:10.1128/aac.00328-23)
Supplement: Supplemental file 1 — Supplemental material. Download aac.00328-23-s0001.pdf, PDF file, 5.3 MB [file aac.00328-23-s0001.pdf]

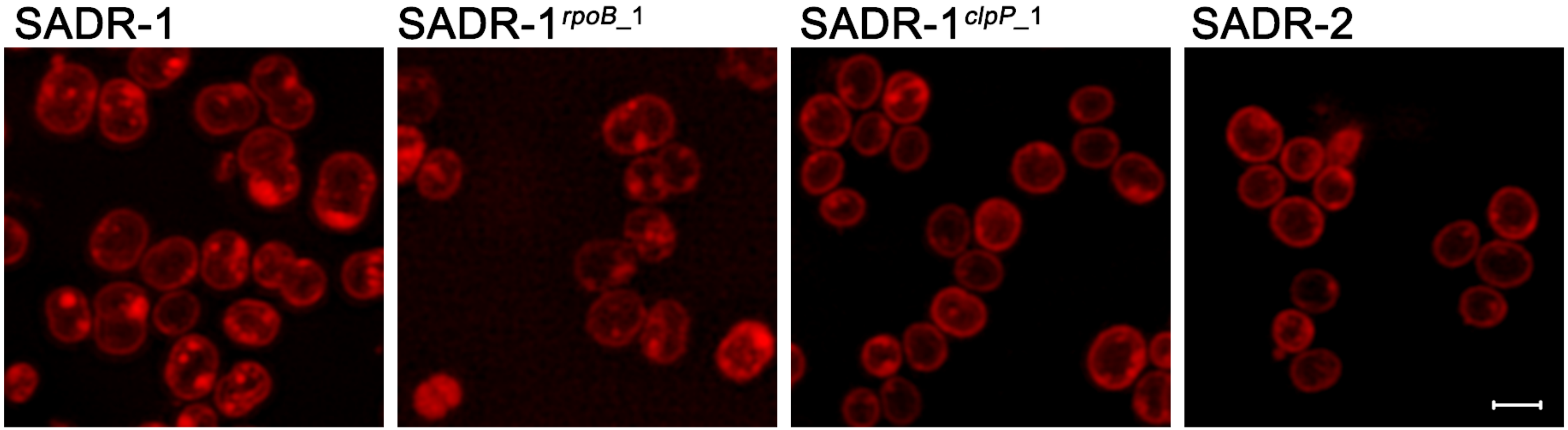

Supplemental Fig. 1. Inactivation of *clpP* mitigates daptomycin-imposed membrane damage. The indicated strains were grown to stationary phase (24 h) and exposed to 20 µg ml^{-1} daptomycin + Ca^{2+} for 60 min at 37°C before staining of membranes with Nile Red and imaging by SR-SIM. Representative pictures are shown from three independent experiments. Scale bar, 1 µm.

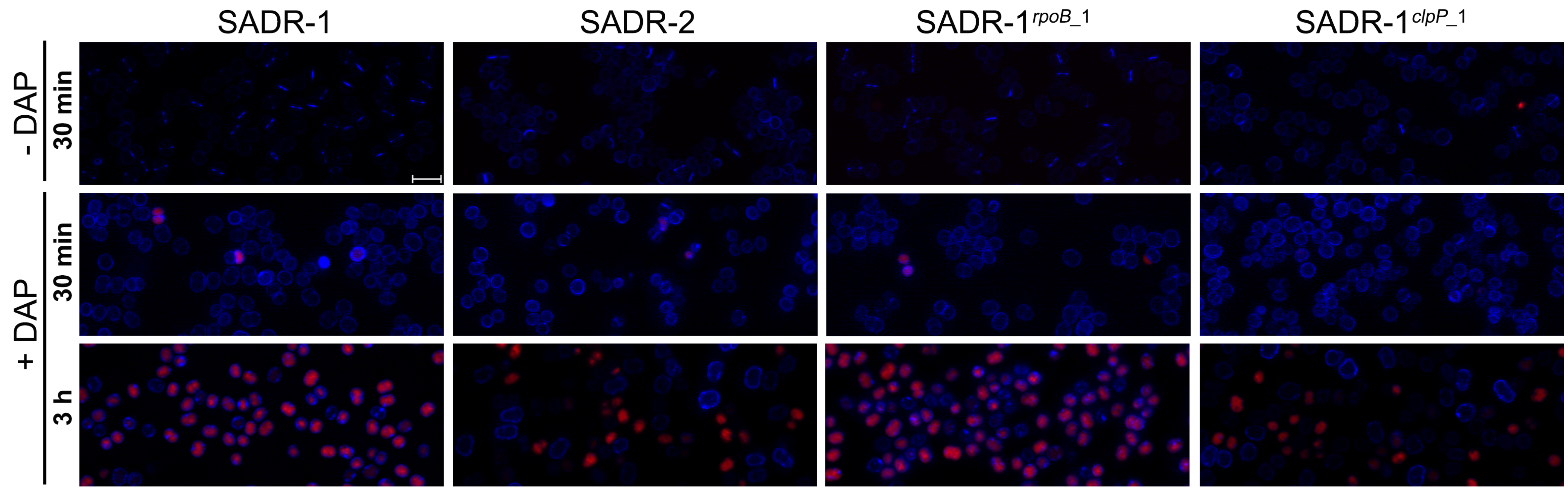

Supplemental Fig. 2. Daptomycin shifts cell wall synthesis from the septal site to the peripheral cell wall. Overnight cultures of the indicated strains were resuspended in TSB (~2 × 10^8 CFU ml-1) and incubated at 37°C ± 20 µg ml^{-1} daptomycin for 30 min or 3 h before imaging with SR-SIM. Prior to SR-SIM, cells were labeled PI (cells with compromised membranes, red), Van-FL (cell wall, green), and HADA (active cell wall synthesis, blue). Overview pictures showing HADA-labeling of the cells shown in Fig. 4 before and 30 min and 3 h following daptomycin exposure.

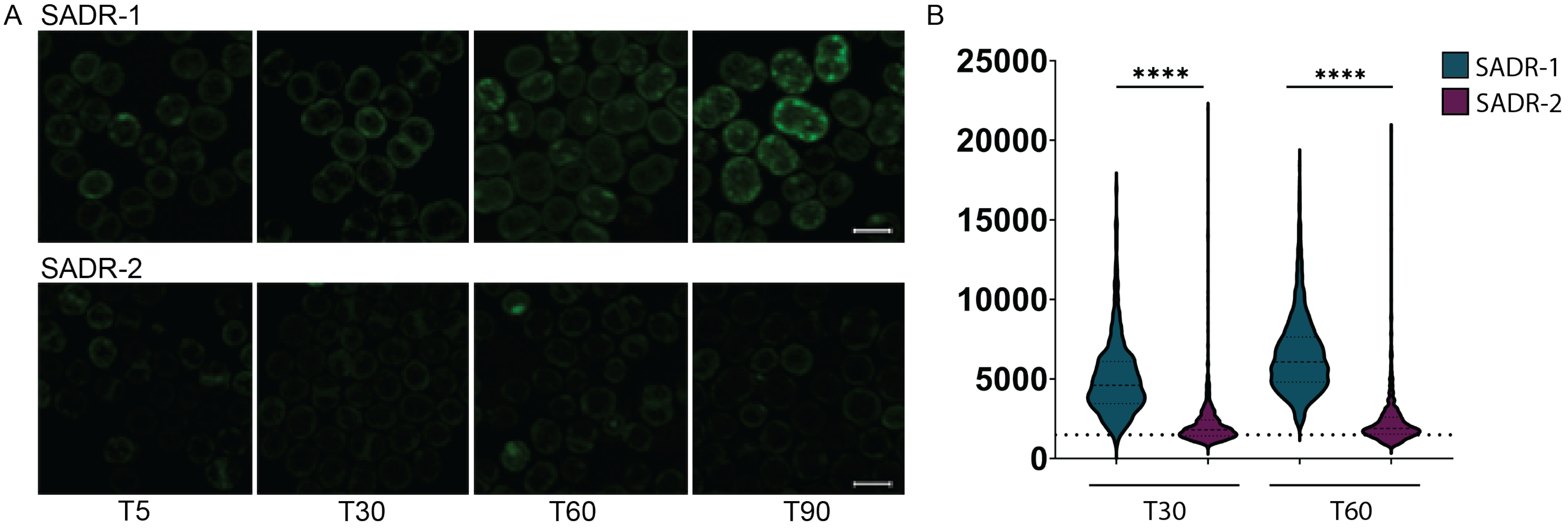

Supplemental Fig. 3. Quantification of Dap-Fl binding in SADR-1 and SADR-2 at different time points. Approximately 2 × 10^8 CFU ml^{-1} of the indicated strains (18 h cultures) were incubated with a mixture of labeled and unlabeled daptomycin to a final concentration of 20 µg ml^{-1} at 37°C before imaging with SR-SIM. Prior to SR-SIM at the indicated time points. Overview pictures showing Dap-Fl-labeling of the cells at the different time points are shown to the left. At T=30 and T= 60, the integrated density was determined in at least 800 random cells for each sample (right side). Measurements were carried out in Fiji and analyzed using GraphPad Prism 9.5 (GraphPad Software LLC). Scale bar, 1 µm.

Supplemental Table 1. Staining and laser specifications used for SR-SIM

Staining	Concentration	Target	Laser	Laser Type	Laser power	Beam splitter	Grating period
Nile Red	5 µg ml^{-1}	Membrane	561 nm	HR Diode – 100 mW	5 % / 100 ms	BP 570-650 + LP 750	34 µm
HADA	250 µM	New PG	405 nm	HR Diode – 50 mW	50 % / 300 ms	BP 420-480 + LP 750	23 µm
TADA	250 µM	New PG	561 nm	HR Diode – 100 mW	20 % / 100 ms	BP 570-650 + LP 750	28 µm
PI	1 µg ml^{-1}	DNA	561 nm	HR Diode – 100 mW	5 % / 100 ms	BP 570-650 + LP 750	23 µm
Van-Fl*	0.8 µg ml^{-1}	Old PG	488 nm	HR Diode – 100 mW	8 % / 100 ms	BP 495-575 + LP 750	28 µm
Dap-Fl**	20 µg ml^{-1}	-	488 nm	HR Diode – 100 mW	8 % / 100 ms	BP 495-575 + LP 750	28 µm

* Van-FL solution comprises a mixture containing equal amounts of vancomycin (Sigma) and a BODIPY FL conjugate of vancomycin to a final concentration of 0.8 µg ml^{-1}.

** Dap-FL solution comprises a mixture containing unlabeled (18 µg ml^{-1}) and Bodipy-FL labeled daptomycin (Dap-FL, 2 µg ml^{-1})
